# Rapid and accurate predictions of perfect and defective material properties in atomistic simulation using the power of 3D CNN-based trained artificial neural networks

**DOI:** 10.1038/s41598-023-50893-9

**Published:** 2024-01-02

**Authors:** Iman Peivaste, Saba Ramezani, Ghasem Alahyarizadeh, Reza Ghaderi, Ahmed Makradi, Salim Belouettar

**Affiliations:** 1 Faculty of Engineering, Shahid Beheshti University, Tehran, Iran; 2 Luxembourg Institute of Science and Technology, 5, Avenue des Hauts-Fourneaux, Esch-sur-Alzette, 4362 Luxembourg; 3 Department of Electrical Engineering, Shahid Beheshti University, Tehran, Iran

**Keywords:** Mechanical properties, Atomistic models, Computational methods

## Abstract

This article introduces an innovative approach that utilizes machine learning (ML) to address the computational challenges of accurate atomistic simulations in materials science. Focusing on the field of molecular dynamics (MD), which offers insight into material behavior at the atomic level, the study demonstrates the potential of trained artificial neural networks (tANNs) as surrogate models. These tANNs capture complex patterns from built datasets, enabling fast and accurate predictions of material properties. The article highlights the application of 3D convolutional neural networks (CNNs) to incorporate atomistic details and defects in predictions, a significant advancement compared to current 2D image-based, or descriptor-based methods. Through a dataset of atomistic structures and MD simulations, the trained 3D CNN achieves impressive accuracy, predicting material properties with a root-mean-square error below 0.65 GPa for the prediction of elastic constants and a speed-up of approximately 185 to 2100 times compared to traditional MD simulations. This breakthrough promises to expedite materials design processes and facilitate scale-bridging in materials science, offering a new perspective on addressing computational demands in atomistic simulations.

## Introduction

In recent years, the field of materials science has witnessed significant advancements, driven by the ever-growing demand for novel materials with superior properties and performance^1–3^. Understanding and predicting the macroscopic behavior of materials are heavily influenced by atomistic structures^4,5^. At the atomic level, materials exhibit a well-organized arrangement of atoms within a crystal structure. However, this orderly arrangement can be disrupted by the presence of defects. In real-world materials, defects like vacancies, dislocations, grain boundaries and voids are unavoidable and have a significant impact on their macroscopic mechanical properties^6^. Understanding how defects affect material properties is crucial for designing materials with improved mechanical performance and durability since the material’s characteristics are significantly influenced by a broad array of defects^7,8^.

To accurately model and understand the behavior of materials, it is essential to employ methods that incorporate the behavior of atoms and their dynamics^9^, and this enables the development of tailored solutions for a wide range of applications spanning from aerospace and automotive industries to electronics and biomedical devices^10^. Due to significant advancements in atomistic computational methods, tools, and the remarkable increase in computer power, scientists and engineers can now effectively simulate material properties and behaviors in specific applications. As a result, this enables them to bypass time-consuming cycles of formulation, synthesis, and testing^11^. One such method is molecular dynamics (MD) simulations, which provide a useful and often indispensable approach for quantitatively studying the properties of materials. By simulating the motion of atoms and molecules under various conditions, MD provides a detailed insight into the complex interactions that govern the mechanical behavior of materials^12^. This level of understanding is essential for predicting and optimizing the performance of materials under various operational conditions. The study of material properties, particularly mechanical properties, in the presence of defects, holds significant importance in materials science. By combining the accuracy of atomistic modeling with the computational power of MD simulations, researchers can unravel the intricate mechanisms underlying defect formation, diffusion, and their impact on material properties, ultimately guiding the development of defect-tolerant materials and improved engineering designs. However, the major challenge associated with nanoscale simulations lies in their computational cost^13,14^. As the size and complexity of the systems under study grow, the number of atoms and interactions to be considered increases exponentially, leading to a substantial demand for computational resources. This, in turn, translates to longer simulation times and higher costs, which can be a significant barrier to the widespread adoption of nanoscale simulations in materials science research and development. Furthermore, The ability to seamlessly connect different lengths and time scales is crucial for understanding the complex behavior of materials across various levels of hierarchy. However, the prohibitive costs associated with extensive simulations in the nanoscale hinder the feasibility of scale-bridging approaches^15^.

In light of these challenges, there is an urgent need for innovative approaches that can bridge the gap between the high accuracy of nanoscale simulations and the practical limitations imposed by their computational cost. Machine learning (ML) has emerged as a promising candidate for addressing this issue, owing to its ability to efficiently learn and generalize complex patterns from large datasets. Besides, ML techniques have shown great potential in capturing complex relationships between various properties, enabling accurate predictions and bridging the gap between different modeling scales. It is interesting to note that ML has been utilized to partially replace costly microscopic simulations^16–18^.

The recent surge in ML applications has led to the development of surrogate computational models based on trained artificial neural networks (tANNs). These models offer an effective alternative to traditional computational methods in various domains, including materials science. By training ANNs on available data, they can capture and learn the underlying patterns and relationships within the data, allowing for fast and accurate predictions. Consequently, tANNs have become valuable tools for accelerating computational processes and reducing reliance on time-consuming simulations. One notable example is the work by Khorrami et al.^19^ who developed tANNs for surrogate modeling of the mechanical response of elasto-viscoplastic grain microstructures. Their trained convolutional neural network (CNN) accurately reproduced the von Mises stress field approximately 500 times faster than numerical solutions of the corresponding initial boundary-value problems based on spectral methods. Similarly, Mianroodi et al.^20^ utilized tANNs to create a surrogate computational model for predicting the stress field in a grain microstructure composed of elastoplastic single crystals. The tANN-based surrogate model significantly outperformed the spectral-based numerical solution of the corresponding periodic initial-boundary value problem (IBVP) in terms of computational speed, achieving an acceleration of up to 8000 times compared to traditional numerical solvers. Furthermore, recently, Eidel et. al^21^ employed trained convolutional neural networks (tCNNs) to link random heterogeneous multiphase materials to their elastic macroscale stiffness, effectively replacing explicit homogenization simulations. The proposed tCNNs model was universal, accounting for various types of microstructures, arbitrary phase fractions, and a wide range of Young’s moduli (1 to 1000 GPa) and Poisson’s ratios (0 to 0.4) for the constituent phases. Several other studies^18,22–24^ have also developed similar surrogate models, further demonstrating the potential of tANNs in predicting mechanical behavior in materials science applications.

In the realm of atomistic simulation, extensive research has been conducted to develop surrogate models for predicting material properties. Furthermore, the materials science community has proposed various representations to describe and characterize crystal structures, as exemplified by studies such as^25–27^. Nyshadham et al.^28^ introduced a rapid surrogate model based on cluster expansion and deep neural networks (DNNs), which effectively estimated material properties like elastic constants, enthalpy, and band-gap with a level of accuracy comparable to ab initio methods. They employed interpolation techniques on a dataset consisting of 10 binary alloys with 10 different species, encompassing all possible fcc, bcc, and hcp structures with up to eight atoms in the unit cell. Their findings indicated that the prediction errors remained within 1.0 meV/atom when increasing the number of simultaneously modeled alloys. Amigo et al.^29^ explored the correlation between multiple material properties and plastic quantities, considering factors such as stoichiometry, sample dimensions, structural properties, temperature, potential energy, and elastic properties. They successfully mapped these input features to plastic quantities like yield stress, flow stress, and ultimate tensile stress using MD simulations. Another notable study by Mianroodi et al.^15^ employed a 2D CNN model to capture atomistic structural images and map them to the corresponding elasticity tensor, which was calculated using molecular statics simulations. This study exemplifies the potential of CNNs in efficiently and accurately predicting structural properties in materials science. Several other studies can be found in^30–35^.

The current body of literature predominantly relies on empirical descriptors or 2D images to establish relationships between atoms and their contributions to overall mechanical properties. However, despite the advancements in surrogate modeling, no previous study has successfully developed a surrogate model that effectively captures the intricate spatial arrangements and coordinates of atoms and incorporates the effects of defects. Addressing these limitations, our research presents a novel approach that aims to fill this gap. By leveraging the power of our proposed method, we expect to demonstrate superior sensitivity in capturing the spatial arrangements of atoms and accurately considering the impact of the defects on material properties. To extend upon previous studies, we utilized 3D CNNs to effectively capture the coordinates of atoms in 3D and calculate their structural properties. This approach offers an efficient method to accelerate atomistic simulations while accounting for the complex chemical and structural features of modern engineering materials. Additionally, we incorporated defects into our investigations to evaluate the sensitivity of this approach. For evaluating our proposed framework, a dataset of atomistic structures with varying numbers of defects was generated, and their elastic constant tensor was calculated using MD simulation. These atomistic structures were then converted into 3D arrays to serve as input for our framework. Subsequently, tANNs with 3D CNNs and multilayer perceptrons were trained using this data and the calculated elastic constant tensor components as a reference dataset.

The evaluation of the trained network demonstrated its ability to capture full atomistic details, including point and volume defects, while achieving remarkable speed-up compared to traditional MD simulation. The obtained results are highly encouraging, as they demonstrate promising accuracy in predicting material properties using tANNs. The root-mean-square error (RMSE) of prediction for all simulation cases is found to be less than 0.65 GPa, indicating the reliability of the neural network’s predictions. Even more impressively, the neural network achieves these accurate results with remarkable efficiency, being considerably faster compared to the traditional MD simulation method. This significant speed-up opens up new possibilities for accelerating materials design processes and enhancing scale-bridging.

Detailed information about the training data, performance, and prediction capability of the tANNs are discussed in the “Results” section, followed by the “Discussion” section. while preparing the atomistic data, network architecture, and training process are presented in the “Methods” section.

## Results

### Atomistic results

To develop a robust ML framework, gathering diverse and precise training data is a crucial initial step. In our supervised learning approach, the dataset for the framework comprises atomistic structures and the corresponding results from MD simulation. To create a comprehensive and varied atomistic structures dataset specifically for BCC Fe, we adopt a systematic approach. Initially, we create perfect crystal structures and then randomly remove atoms from their respective positions within the crystal structure, introducing controlled vacancy sites that form the basis of our subsequent analysis. It’s important to emphasize that the progression of vacancy sites followed a precise sequence, starting from 0% and increasing in increments of 0.1% until reaching 5%. This systematic approach ensured the generation of a diverse and well-structured dataset, enabling the ML framework to effectively capture the behavior of BCC Fe with varying vacancy concentrations. To ensure the generation of a rich and robust dataset, we leveraged the capabilities of Pymatgen^36^, an open-source Python library specifically designed for materials analysis. Utilizing the powerful features of Pymatgen, we created atomistic structures that precisely reflected the crystal lattice’s structure and geometry. To enhance the statistical significance of the dataset and facilitate a comprehensive analysis, we performed 220 simulations for each specific vacancy concentration. While the concentration of vacancies remains fixed during each simulation, the positions of the removed atoms are determined randomly. This deliberate randomness not only provides a diverse set of atomistic configurations but also exponentially increases the number of simulations. Consequently, the dataset grows in size, providing a larger and more representative sample for subsequent ML processes. Overall beginning from 0 to 5% vacancy with incrimination of 0.1%, also repeating creating for each specific percent 220 times leads to the creation of 50 × 220 = 11000 atomistic structure. The size of the supercell is set as (Lx, Ly, Lz) = (16, 16, 16) × a0, where a0 represents the lattice constant of BCC Fe, measuring 2.856 Å. By employing this approach, the supercell encompasses a total of 8192 atoms. Also, the periodic boundary conditions are adjusted to the simulated supercell (simulation box). The utilization of periodic boundary conditions allows us to mimic an infinite crystal lattice by seamlessly connecting the edges of the supercell. This approach effectively eliminates boundary effects, enabling us to study the behavior of the BCC Fe crystal lattice under different vacancy concentrations with enhanced accuracy and reliability. By combining the principles of random vacancy generation, precise atomistic structure creation using Pymatgen, and the repetition of simulations for statistical significance, we ensured the creation of a coherent and cohesive atomistic structures dataset, poised to support our ML analysis effectively. Each structure was then subjected to MD simulation and the embedded atom method (EAM) potential developed by Olsson^37^. The chosen EAM potential has been extensively validated and demonstrates high accuracy in predicting the mechanical characteristics of Fe. This is also corroborated by the results obtained by ours and presented in Table 1. Our results also agree with^38^ for Fe with point defects.

**Table 1:** Comparison of experimental values and simulation of mechanical properties of BCC Fe.

	Experimental	LAMMPS
$C_{11}$ cubic	230^39^	224.988
$C_{12}$ cubic	138^40^	137.623
$C_{44}$ cubic	121^40^	120.027
Bulk modulus	166^41^	166.435
Shear modulus	77.5^41^	79.728
Elasticity modulus	200^41^	206.252
Poisson ratio	0.291^41^	0.2934
Vacancy formation energy	$1.7\pm 0.2$^42^	1.69

In order to make the atomistic structures suitable for the input of 3D CNNs, specific preprocessing steps were applied, which will be detailed in the Methods section. On the other hand, the output for our neural networks is the elastic constant tensor, which is obtained through MD simulations.

### Performance evaluation of ML predictions

In the initial assessment, we subjected the tANNs to a comprehensive evaluation using 1500 randomly generated cases from the test dataset. It is important to note that these specific data points were entirely excluded from the training and validation processes. The root-mean-square error (RMSE) obtained by the trained network on the test dataset was approximately 0.42 GPa. This level of error is comparable to the RMSE of 0.65 GPa observed on the validation dataset. These values correspond to around 0.2%, 0.3%, and 0.4% relative error in predicting $C_{11}$, $C_{12}$, and $C_{44}$ of single-crystalline iron, respectively. Notably, such high accuracy has not been seen in previous models, and it holds significant promise for practical applications in atomistic simulation. The ability to predict material properties with such precision opens up new possibilities for advancing materials science and engineering, enabling more efficient and reliable design processes for a wide range of applications. Furthermore, we conducted an examination of additional select cases, which are voids, to assess and interpret the network’s performance. It is worth highlighting that these particular cases were not included in either the training or validation datasets.

### Vacancy concentration effects

At any given temperature, a material possesses an equilibrium concentration of vacancies, representing the ratio of vacant lattice sites to those occupied by atoms. The creation of vacancies demands a net input of energy, as it involves breaking existing bonds, but some energy is recovered when new bonds form with other atoms on the surface^43^. The concentration of vacancies within the material is subject to fluctuations due to several factors, such as temperature variations, defect sources, quenching, and formation energy. To investigate and simulate the impact of vacancy concentration on materials, various computational methods are available, with MD being a commonly employed approach^44^.

**Figure 1:**
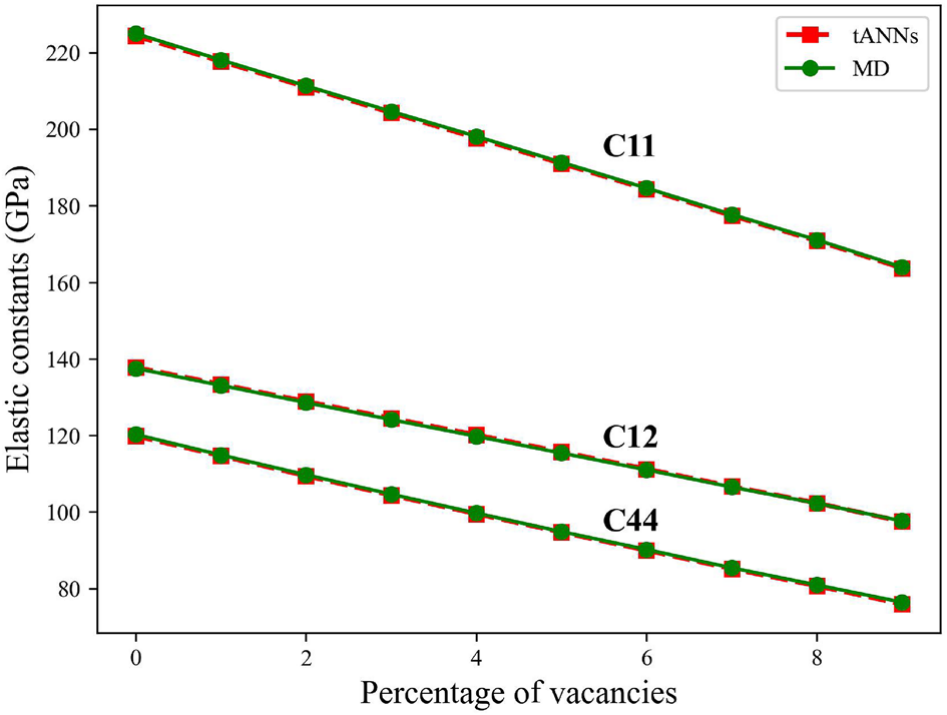
Comparative analysis of elastic constants calculation and prediction Using MD and tANNs for Varying Vacancy Percentages. The figure showcases the remarkable performance of our framework across different vacancy percentages, highlighting its accuracy even beyond the training data range.

Earlier, we presented the capabilities of our ML framework in predicting the mechanical properties of materials containing vacancy concentrations ranging from 0 to 5%, achieving a high accuracy level with a mean relative error of just 0.3%. To further illustrate the generalizing capabilities of the ML framework, we extended the application to structures with higher vacancy concentrations. The results, as depicted in Fig. 1, demonstrate the framework’s excellent performance even for data beyond the training range.

Remarkably, the ML framework, although trained on data with up to 5% vacancies, showcased impressive performance on data with higher vacancy concentrations, highlighting its robustness and adaptability in dealing with scenarios it has not encountered during training.

To conduct a thorough comparison between the outcomes produced by the tANNs model and MD simulations, we embarked on an additional experimental phase. In this phase, we systematically generated a set of atomistic structures characterized by varying vacancy concentrations, ranging from 0 to 10. These structures were subsequently input into the tANNs, which in turn provided predictions for their respective mechanical properties. Simultaneously, we carried out MD simulations for these same atomistic structures using the LAMMPS. The outcomes of both approaches, namely the predictions from tANNs and the numerical results derived from MD simulations, were compiled and are presented in Table 2. In addition, to gauge the accuracy of tANNs, we calculated the relative error between the two sets of results. This relative error serves as a vital indicator of how closely the ML model aligns with the MD results. This observation underscores the remarkable agreement and consistency between the predictions made by tANNs and the precise numerical outcomes generated through MD simulations. Furthermore, the consistent maintenance of a low relative error when applied to data beyond the training set serves as a compelling testament to the exceptional capability of ML in terms of generalization.

We have extended our examination to encompass additional mechanical properties that can be derived from the elastic constants, the elastic constants, as described in refs^45,46^, and elucidated in the methods section. Comprehensive mechanical behavior regarding these derived properties can be found in the supplementary Information.

**Table 2:** Comparison of mechanical properties predicted by tANNs model and MD simulations for atomistic structures with varying vacancy concentrations (0 to 10).

Percentage of vacancy	MD values (GPa)			tANNs values (GPa)			Relative error%		
0	$C_{11}$	$C_{12}$	$C_{44}$	$C_{11}$	$C_{12}$	$C_{44}$	$C_{11}$	$C_{12}$	$C_{44}$
	224.988	137.805	120.265	224.357	137.533	119.855	0.28	0.197	0.34
1	$C_{11}$	$C_{12}$	$C_{44}$	$C_{11}$	$C_{12}$	$C_{44}$	$C_{11}$	$C_{12}$	$C_{44}$
	218.123	133.369	114.907	217.731	133.09	114.673	0.179	0.209	0.203
2	$C_{11}$	$C_{12}$	$C_{44}$	$C_{11}$	$C_{12}$	$C_{44}$	$C_{11}$	$C_{12}$	$C_{44}$
	211.341	128.902	109.725	211.017	128.59	109.436	0.146	0.242	0.263
3	$C_{11}$	$C_{12}$	$C_{44}$	$C_{11}$	$C_{12}$	$C_{44}$	$C_{11}$	$C_{12}$	$C_{44}$
	204.654	124.494	104.653	204.293	124.13	104.391	0.176	0.499	0.25
4	$C_{11}$	$C_{12}$	$C_{44}$	$C_{11}$	$C_{12}$	$C_{44}$	$C_{11}$	$C_{12}$	$C_{44}$
	198.158	120.157	99.7165	197.725	119.787	99.5237	0.218	0.307	0.323
5	$C_{11}$	$C_{12}$	$C_{44}$	$C_{11}$	$C_{12}$	$C_{44}$	$C_{11}$	$C_{12}$	$C_{44}$
	191.359	115.692	94.8591	191.037	115.395	94.702	0.168	0.256	0.156
6	$C_{11}$	$C_{12}$	$C_{44}$	$C_{11}$	$C_{12}$	$C_{44}$	$C_{11}$	$C_{12}$	$C_{44}$
	184.617	111.233	90.2093	184.328	111.017	89.9814	0.156	0.194	0.252
7	$C_{11}$	$C_{12}$	$C_{44}$	$C_{11}$	$C_{12}$	$C_{44}$	$C_{11}$	$C_{12}$	$C_{44}$
	177.742	106.69	85.4007	177.421	106.515	85.1471	0.18	0.164	0.296
8	$C_{11}$	$C_{12}$	$C_{44}$	$C_{11}$	$C_{12}$	$C_{44}$	$C_{11}$	$C_{12}$	$C_{44}$
	171.074	102.336	80.9335	170.833	102.256	80.6902	0.141	0.078	0.3
9	$C_{11}$	$C_{12}$	$C_{44}$	$C_{11}$	$C_{12}$	$C_{44}$	$C_{11}$	$C_{12}$	$C_{44}$
	163.982	97.5064	76.44	163.712	97.6686	75.9397	0.164	0.166	0.654
10	$C_{11}$	$C_{12}$	$C_{44}$	$C_{11}$	$C_{12}$	$C_{44}$	$C_{11}$	$C_{12}$	$C_{44}$
	154.345	91.9722	70.4095	154.079	91.812	70.187	0.172	0.174	0.316

### Void effects

Accumulation of vacancies can result in the formation of voids or pores within the material, which subsequently leads to a reduction in its overall strength and ductility^47^. Voids in metallic materials typically arise due to an imbalanced atomic diffusion flux, except for those formed as a result of manufacturing defects^48^. Voids can also give rise to failure, crack initiation, anisotropy, and moisture penetration^4,48,49^. Consequently, studying the void impact on mechanical properties is essential for ensuring the quality and performance of materials in various applications. The reduction of strength due to voids can be quantified through MD.

**Figure 2:**
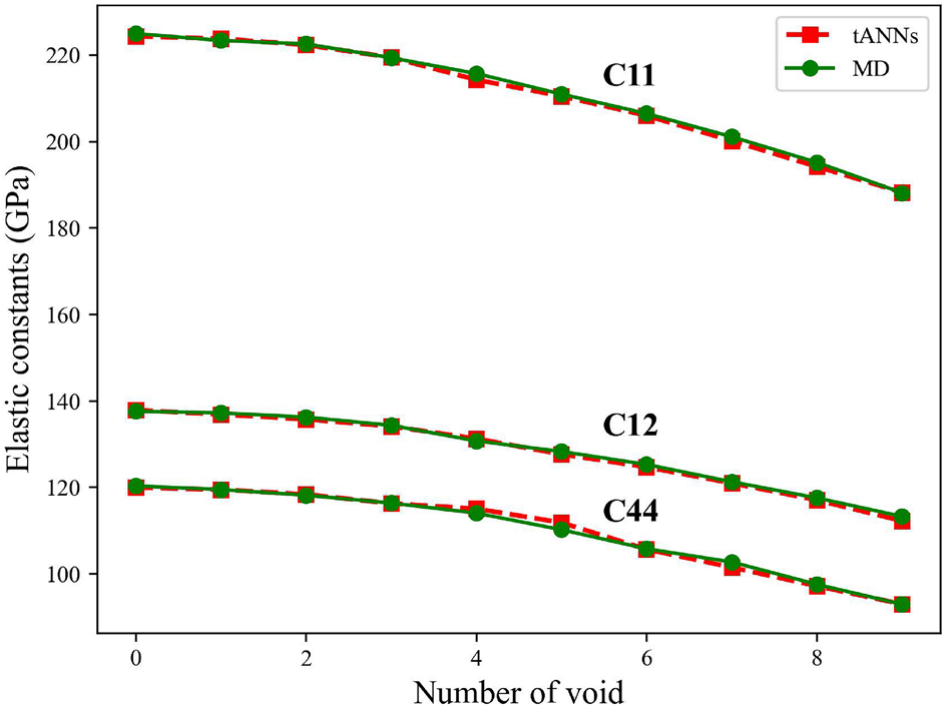
Comparative analysis of elastic constants calculation and prediction Using MD and tANNs for varying number of voids. The figure showcases the remarkable performance of our framework across different types of defects.

In the preceding section, we demonstrated the capability of our ML framework to accurately predict material properties when dealing with random vacancies. In this section, our focus shifts to investigating the framework’s effectiveness in predicting the material’s behavior with randomly distributed voids within the crystals. Figure 2 showcases the impact of the number of voids on the elastic constants.

To generate each void, a random atom and its surrounding atoms (eight in total, considering the coordination number of 8 for BCC crystal structures) were removed, creating a realistic representation of voids in the material. It is worth noting that voids were not included in the training data. Despite this, our network exhibits remarkable performance in predicting the behavior of BCC Fe when random voids are introduced. This capability demonstrates the robustness and generalizing capacity of our framework beyond the scope of the training.

### Computational speed

The above-presented results reveal a high level of accuracy achieved by neural networks in predicting the elastic constant tensor based on the input atomistic structures. To assess the computational speed improvement achieved by the network compared to conventional forward simulations, we conducted experiments using a different number of processors of an AMD Ryzen Threadripper 3970x, operating at 2.2 GHz, for both MD simulation and obtaining the neural networks’ output.

**Figure 3:**
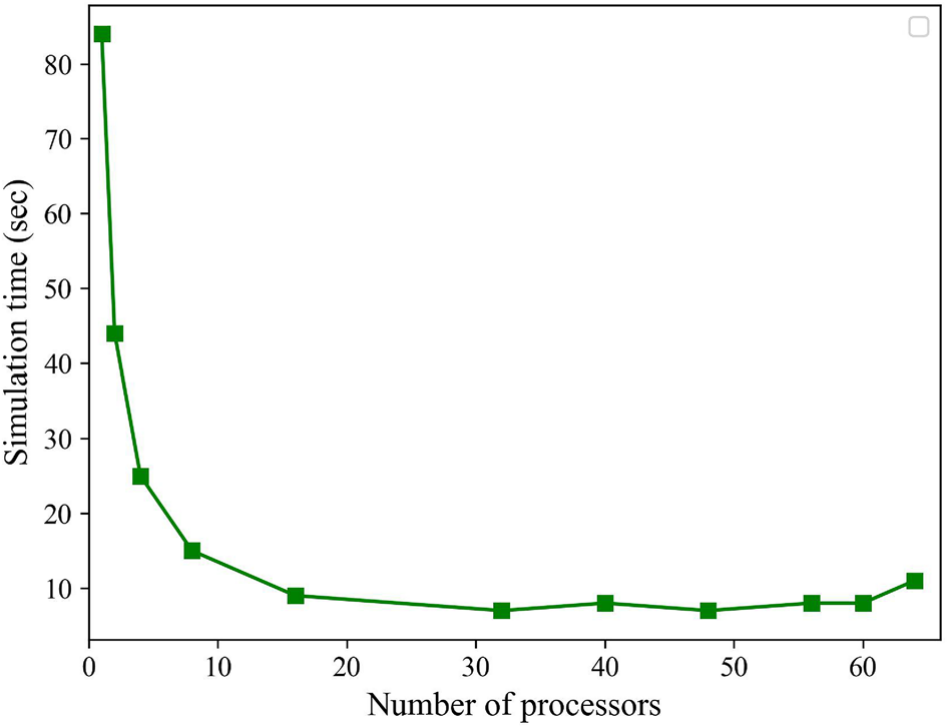
Effect of processor count on computation time for a defect-free perfect structure. The graph depicts how computation time decreases with an increasing number of processors, yet it plateaus at a lower limit of 7 s, indicating a minimum achievable computation time.

The neural networks calculate the elastic constant tensor in an impressively short time of just 38 ms, while LAMMPS, depending on the number of processors used, takes between 84 to 7 s for the same computation, as demonstrated in Fig. 3. Notably, the computation time for predicting the elastic constant tensor using the trained networks remains consistent irrespective of the complexity of geometry, thermodynamic conditions, or the number of processors used. In contrast, MD simulation time is influenced by various factors, such as time steps, ensembles, force fields, and other parameters, making it more variable and context-dependent. To cope with the computational demands of MD simulations, parallel computing techniques are commonly employed, dividing the simulation into smaller tasks processed simultaneously on multiple processors or computing nodes to reduce overall simulation time and increase efficiency. However, increasing the number of processors may not always lead to improved computation time due to communication overhead, load imbalance, or other factors. Figure 3 illustrates the computation time based on the number of cores for a perfect structure (without defects), showing the best computational time achieved at around 7 s. This indicates a limit for computation time, yet our framework managed to achieve a remarkable speedup. The remarkable difference in processing time between the neural networks and traditional methods, as previously demonstrated, signifies the potential of neural networks for nano-scale calculations. Specifically, our framework allows for computations that are approximately 185 to 2100 times faster while maintaining high accuracy. This substantial speedup opens up exciting possibilities for efficient and reliable nano-scale simulations, enabling researchers and engineers to explore and analyze complex materials and phenomena with unprecedented speed and precision.

It’s important to mention that the reported speedup is an estimate, as both the neural network and MD calculations could potentially be optimized further for greater efficiency. For instance, in this study, we concentrated solely on the elastic constant tensor, but the neural network has the capability to handle more intricate and time-consuming parameters, broadening its potential for diverse applications beyond this specific task. The ability to explore more complex material properties with high efficiency further highlights the versatility and potential impact of our proposed framework.

## Discussion

The landscape of advanced materials and the products derived from them has evolved into a realm of profound complexity. In the present day, these structural materials exhibit a multifaceted character, incorporating numerous crystals, defects, and phases. Accelerating computer simulations holds the key to expediting scientific discoveries, particularly in the realm of materials science, where numerous real-world applications encompass a wide range of length and time scales.

This investigation introduces a novel method that harnesses the power of ML techniques to address computational challenges in achieving precise atomistic simulation results within the field of materials science. Concentrating on MD in the context of materials behavior at the atomistic level, the study showcases the potential of tANNs as surrogate models. These tANNs adeptly capture intricate patterns from meticulously curated datasets, enabling rapid and precise predictions of materials properties. A noteworthy advancement is the utilization of 3D CNNs to incorporate atomistic intricacies and defects into predictions, presenting a significant stride beyond prevailing 2D image-based or descriptor-based methodologies.

The development of a new tANNs framework in our study unfolds in three distinct stages, each contributing to its overall effectiveness. These steps are as follows: I) Dataset generation. In the initial phase, our focus was on constructing a comprehensive and representative dataset. This dataset comprises atomistic structures alongside their corresponding simulation results obtained through MD simulations. To streamline this data collection process, we devised a Python script. This script efficiently generates atomistic structures featuring various defects and seamlessly interfaces with the LAMMPS simulation software. The generated data is then systematically stored in an array. This entire data generation process operates iteratively, ensuring a robust and diverse dataset. II) Data transformation. The subsequent stage revolves around the transformation of these atomistic structures to a format suitable for consumption by 3D CNNs. Traditionally represented as columns of x, y, and z coordinates, these structures are adapted into a 4D tensor format, optimizing them for further processing within the neural networks. III) Creating, training, and optimizing neural networks. The development of neural networks for detecting local properties and aggregating information from complex atomistic structures, particularly in the presence of defects, demands careful consideration of network architecture. In this context, CNNs emerge as a highly suitable choice due to their inherent capability to efficiently process spatial information. The efficiency of our trained CNN substantiates this choice, as it demonstrates the ability to accurately predict the elasticity tensor of entirely novel structures. Remarkably, these structures were not part of the original training dataset, highlighting the network’s capacity for generalization and its robustness in handling previously unseen data. In the end, the neural networks undergo extensive training and optimization. This crucial phase fine-tunes the networks’ parameters and architecture to achieve the best possible results, ensuring that our framework delivers accurate predictions and maintains its efficiency gains in atomistic simulations.

The research culminates in a remarkable achievement and the trained 3D CNNs along with MLP display impressive accuracy, capturing full atomistic details including points and volume defects. To evaluate the efficiency of our proposed framework, we conducted tests to predict the elastic constants of BCC Iron (Fe) in the presence of point and volume defects. The results underscore the framework’s proficiency in predicting elastic constants within atomistic simulations, achieving an impressive RMSE of less than 0.65 GPa. Notably, this predictive accuracy is coupled with a substantial acceleration in computational speed, with our approach demonstrating a speed enhancement ranging from approximately 185 to 2100 times faster when compared to conventional MD simulations. This not only attests to the accuracy of our model but also underscores its potential to enhance the accuracy of surrogate models for atomistic-scale simulations.

The study underscores the synergy between ML and MD, demonstrating the potential of tANNs to serve as effective surrogates for complex and time-consuming simulations. Moreover, the incorporation of 3D CNN advances the accuracy and efficiency of predictions, enabling the consideration of atomistic details and defects, previously unattainable by conventional methodologies.

## Methods

This section provides a comprehensive description of the methodology employed in this study, covering the preparation of atomistic data, the neural network architecture, and the training process of the algorithm. To begin, a dataset of atomistic structures is meticulously constructed, following which specific defects are introduced into these structures. Subsequently, the numerical values corresponding to the properties of these modified structures are computed. Moving forward, these computed properties are utilized as outputs, while the structural characteristics of the atomistic configurations serve as input features for the neural network. In essence, the neural network is designed to take the structures as inputs and predict the corresponding properties as outputs. By delineating the intricate process of dataset creation, defect incorporation, property computation, and network utilization, we establish a solid foundation for our subsequent analyses and showcase the seamless integration of atomistic data and ML techniques in this research endeavor.

### Atomistic structures dataset preparation

The effectiveness of ML methods is mainly influenced by three key factors: the size, quality, and diversity of the training data. These aspects play a critical role in determining the performance and generalization capabilities of ML models^50^.

To establish a relatively large, comprehensive, and diverse atomistic structures dataset, tailored specifically for BCC Fe, we employed Pymatgen, a powerful materials python library. Specifically, the “structure” class within the “core” package is used to efficiently generate and manipulate periodic atomistic structures with precision, ensuring the dataset’s completeness and diversity. The “data” class with the “lammps” package is also used to generate LAMMPS data files from generated structures.

We systematically established a comprehensive and diverse atomistic structures dataset for BCC Fe by creating perfect crystal structures and progressively introducing controlled vacancy sites, ranging from 0 to 5% with 0.1% increments. This methodically sequenced approach ensured the dataset’s diversity and structure. For robust statistical significance, we conducted 220 simulations for each vacancy concentration, employing randomness in atom removal positions, thereby exponentially expanding the dataset size to 11000 atomistic structures. The supercell size, defined by periodic boundary conditions, was set at (Lx, Ly, Lz) = (16, 16, 16) × a0, encompassing 8192 atoms. This strategy eliminated boundary effects, enhancing accuracy. By integrating random vacancy generation, precise atomistic structure creation using Pymatgen, and repetitive simulations, we established a cohesive dataset. Each structure underwent MD simulation to compute its elastic constant tensor, crucial for neural network output and predictive analyses. This comprehensive methodology bridges atomistic structures and ML processes, forming the foundation for our framework’s evaluation and predictions.

### MD simulation

In the subsequent step, the atomistic structures generated in the previous section were subjected to MD calculations to determine their elastic constants which determine mechanical properties. For the evaluation of our framework, we specifically focused on BCC Fe. To facilitate these calculations, we employed the LAMMPS code^51^, a widely-used MD software package.

By leveraging the capabilities of the LAMMPS code and employing the validated EAM potential, we conducted MD calculations on the generated atomistic structures. This approach enabled us to extract crucial mechanical properties, such as elastic constants that can be used to obtain bulk modulus, shear, and young’s modulus, which are indicative of the material’s mechanical behavior. Through these calculations, we obtained a comprehensive understanding of the mechanical response of the BCC Fe crystal lattice under different vacancy concentrations.

At first, the atomistic sample undergoes relaxation to a stress-free state employing the conjugate gradient method^52^. This method is an algorithm that iteratively searches for the minimum of the function by moving in directions that are conjugate to each other, which means each search direction is orthogonal to the previous ones in the space defined by the function’s Hessian matrix. After that, Firstly, the simulation box was brought into equilibrium using two distinct ensembles: the NVE ensemble and the Langevin (stochastic) thermostat ensemble. The timestep for both these ensembles was considered .001 ps. This choice is based on a balance between accuracy and computational efficiency for the simulations. The system then ran for 10000 number of timesteps. This process ensured that the system reached thermal equilibrium which is considered 300K in our study. Once the equilibration phase in the desired temperature was completed, we introduced small deformations to the simulation box along the positive and negative axes of the X, Y, and Z directions. These deformations were characterized by different strain values, ranging from 0.0001 to 0.5. These controlled deformations allowed us to study the system’s response to various levels of strain. Based on the obtained results, a strain value of 0.01 was selected for calculating the elastic constants. This value was determined to be optimal for accurately capturing the mechanical behavior of the system. Following each deformation, the simulation box was once again subjected to the NVE ensemble and the Langevin thermostat with the same timestep. We raised the number of timesteps to 30000 in this stage to ensure that the system remained at the desired temperature and achieved thermal equilibrium. To establish the relationship between stress and strain for small displacements, we employed Hook’s Law. Utilizing the tensorial form of Eq. (1) in Voigt notation, we expressed the stress-strain relationship. In this notation, both stress and strain are represented as three-component tensors, denoted as $C_{ij}$, where *C* represents the elastic constant.

1 $$\begin{aligned} \sigma _i=\sum _{1}^{6}C_{ij} \varepsilon _{ij} \end{aligned}$$

In certain elasticity studies, it is advantageous to express strains in terms of stresses. This leads to the formulation of Eq. (2), where $S_{ij}$ represents elastic compliance constants:

2 $$\begin{aligned} \varepsilon _i = \sum _{1}^{6} S_{ij} \sigma _j \end{aligned}$$

By employing the principles of continuous elastic theory, Eqs. (1) and (2) can be adapted for triclinic crystals, resulting in 16 independent constants. However, in the case of cubic crystals, the equations are simplified due to geometric symmetry, and each tensor can be expressed using only three independent constants. Generally, these reduced equations are written as follows:

3 $$\begin{aligned} C= & {} \begin{bmatrix} C_{11} &{} C_{12} &{} 0 &{}0 \\ C_{21} &{} C_{22} &{} 0 &{} 0 \\ 0 &{} 0 &{} 0 &{} 0\\ 0 &{} 0 &{} 0 &{} C_{44} \end{bmatrix} \end{aligned}$$

4 $$\begin{aligned} S= & {} \begin{bmatrix} S_{11} &{} S_{12} &{} 0 &{}0 \\ S_{21} &{} S_{22} &{} 0 &{} 0 \\ 0 &{} 0 &{} 0 &{} 0\\ 0 &{} 0 &{} 0 &{} S_{44} \end{bmatrix} \end{aligned}$$

Equations (5)–(7) reveal the relationships between elastic and compliance constants:

5 $$\begin{aligned} S_{11} =\, (C_{11}+ C_{12})/(C_{11}-C_{12} )(C_{11}+2C_{12} ) \end{aligned}$$

6 $$\begin{aligned} S_{12} =\, (- C_{12})/(C_{11}-C_{12} )(C_{11}+2C_{12} ) \end{aligned}$$

7 $$\begin{aligned} S_{44} =\, 1/C_{44} \end{aligned}$$

The mechanical properties of cubic systems, including the bulk modulus, shear modulus, and young’s modulus, can be calculated using the Voigt–Reuss–Hill scheme. This scheme employs the following Eqs. (8)–(12), where H, V, and R represent the Hill, Voigt, and Reuss approximations, respectively:

8 $$\begin{aligned} B_H = B_V = B_R = (C_{11}+ 2C_{12})/3 \end{aligned}$$

9 $$\begin{aligned} G_H =\, (G_V+ G_R)/2 \end{aligned}$$

10 $$\begin{aligned} G_V =\, (C_{11}- C_{12}+3C_{44})/5 \end{aligned}$$

11 $$\begin{aligned} G_R =\, (5(C_{11}- C_{12})C_{44})/(4C_{44} +3(C_{11}- C_{12})) \end{aligned}$$

12 $$\begin{aligned} E_H =\, (9B_H \times G_H)/(3B_H+G_H ) \end{aligned}$$

### Input and output generation for convolutional neural network

The position of atoms within the supercell is conveniently represented by a 2D array, where the number of rows corresponds to the total number of atoms. In this 2D array, each atom’s position is described using three columns, denoting its coordinates along the x, y, and z axes within the lattice structure. To facilitate this conversion from the atomistic structures to a 2D array format, we utilized the class structure and its attributes in the Pymatgen library.

To account for the presence of vacancies, we introduced an additional fourth column in the array. If an atom is present, the corresponding entry in the fourth column is assigned a value of 1. Conversely, if an atom has been removed to create a vacancy, the entry is set to 0. By incorporating this information, we effectively capture the presence or absence of atoms within the lattice structure.

**Figure 4:**
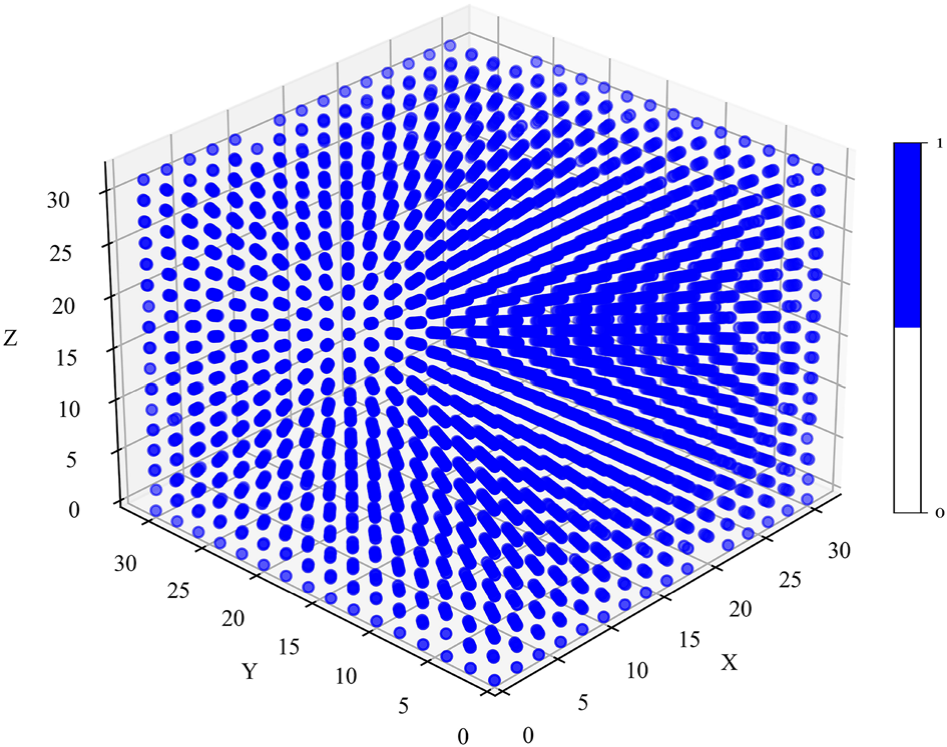
Visualization of Atomistic Structures in a 3D Array: Atoms represented by ’1’ and vacancies by ’0’ within a 3D grid.

**Figure 5:**
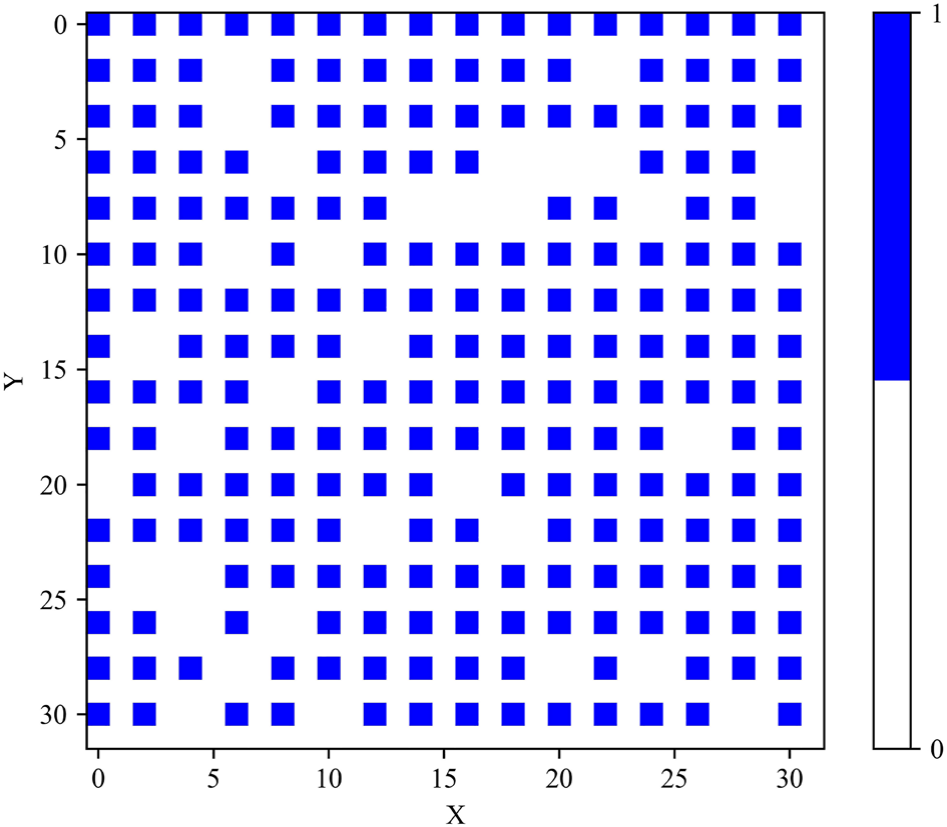
Visualization of Atomistic Structures in a 2D Array (z = 0): Atoms represented by ’1’ and vacancies by ’0’ within a 3D grid.

To ensure consistency and convenience, we normalized the positions of the atoms along the x, y, and z axes. Specifically, we divided the position values in the first three columns by half of the lattice constant. This transformation yields integer values for each column, facilitating positioning them into a 3D array. Subsequently, we created an empty 3D array with dimensions (32, 32, 32) to accommodate the transformed data from the 2D array. By populating this 3D array with the corresponding atom and vacancy information, we obtain a comprehensive representation of the system. In this representation, atoms are represented as 1 in the grid, while vacancies are denoted by 0. Overall, this conversion process enables us to effectively transform atomistic structures into a convenient 3D array format, facilitating further analysis and calculations within our research framework. Figure 4 displays the arrangement of atoms within a 3D array. To provide a clearer visualization, we further depict the first layer along the z-axis (z = 0) in Fig. 5.

Once the structures have been generated and transformed into a 3D array format, they serve as input for the CNN layers. The CNN processes the input data and produces corresponding outputs, including the elastic constant tensors. By utilizing the power of CNNs, we can effectively extract meaningful features from the input structures and predict important mechanical properties. contributing to a deeper and much faster understanding of the material’s performance on the nanoscale.

### Neural network architecture and its training

**Figure 6:**
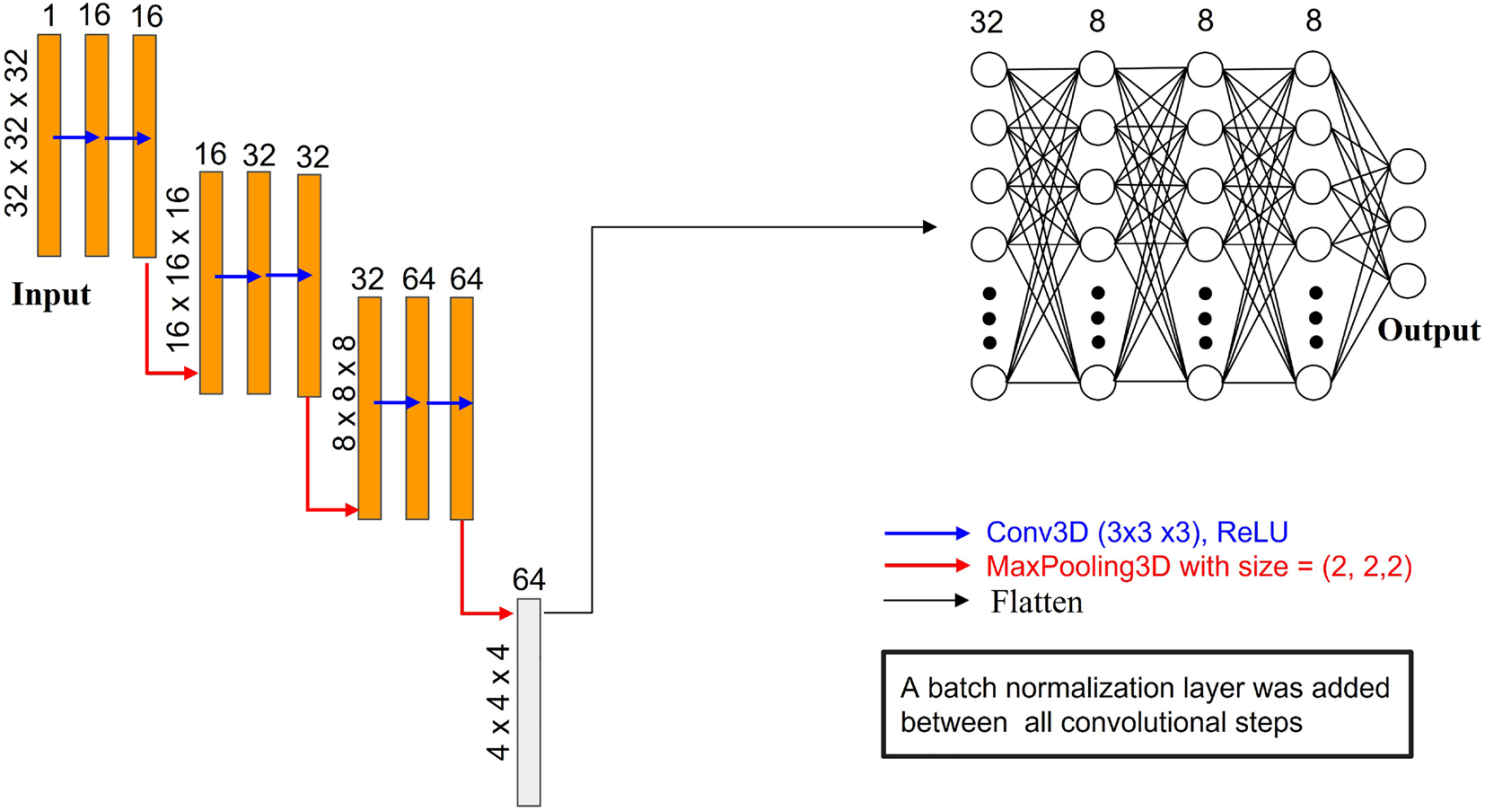
The neural network structure employed in this research.

Neural networks are powerful computational systems consisting of interconnected neurons that collaborate to perform complex tasks such as image recognition, pattern recognition, natural language processing, and prediction. These networks learn from data by training on a set of input-output pairs, enabling them to capture underlying patterns and relationships. Training involves optimizing the weights and biases of the neurons through iterative optimization algorithms like backpropagation. The architecture of a neural network refers to the organization of its neurons and their connections. Different types of neural network architectures exist, including feedforward, recurrent, convolutional, and deep neural networks. The choice of architecture depends on the specific task at hand and the characteristics of the input data. In our research, we leverage the power of CNNs along with deep neural networks due to their advantageous features. CNNs excel in processing arrays efficiently, achieving high accuracy rates, and demonstrating robustness to noise. They are also capable of rapidly learning complex features, generalizing well to unseen data, and handling large quantities of intricate data in array form^53^. During the training process, the hyperparameters of the neural network need to be carefully tuned to optimize performance. Hyperparameters include parameters such as learning rate, batch size, and network depth, among others. These choices greatly influence the network’s ability to learn and generalize from the training data. To build our neural network, we leverage the capabilities of the PyTorch library^54^. PyTorch provides a rich set of operations and functions that facilitate the construction and training of neural networks. In our implementation, we utilize the sequential model, which allows us to create a linear stack of layers that form the complete network architecture. The initial layers of our network are of the convolutional type. These convolutional layers play a crucial role in transforming the input objects, which are represented as arrays, and extracting meaningful features from them. By applying convolutional operations, the network can effectively capture local patterns and spatial relationships within the input data. This enables the network to learn hierarchical representations of the features, leading to improved performance in subsequent layers. Following the convolutional layers, we proceed to flatten the data, preparing it for mapping to the desired outputs, which in our case are the mechanical properties. To achieve this, we utilize dense layers in our neural network architecture. Dense layers, also known as fully connected layers, serve as a bridge between the extracted features from the previous convolutional layers and the final output predictions. The flattened data from the convolutional layers are fed into these dense layers, where each neuron is connected to every neuron in the previous layer. The architecture of the network is shown in Fig. 6.

#### Neural network architecture

Neural networks can vary in terms of their fundamental building blocks, the organization of these blocks within the layers, the way they are connected, and the characteristics of the loss functions used. The left side of the neural network used in this study (in orange color in Fig. 6) is the encoder component of the U-Net architecture^55^, which prior research has demonstrated is highly effective for accurately predicting microstructure evolution using phase-field simulations^16^. Within each of the three encoder stages, the data undergoes processing through two convolution layers. The padding used for each convolutional layer is set to “same” which ensures that the dimensions of the output data match those of the input data. convolutional layers include a set of filters (kernels). Note that the convolution operation is a sliding window technique that applies the filter over the image, element-wise multiplication between the filter and the input pixels is then accumulated and fed into a single neuron in the next layer. By arranging convolution layers in a stacked manner within each stage of the encoder, it enables the effective correlation of significant features present in the input data. As stated previously, this work is implemented based on 3D operations, So we use a kernel size of (3 × 3 × 3). Increasing the number of kernels in the network design generally results in improved network performance. In other words, the network’s ability to detect features is greatly enhanced^56^. The other component of these layers is the activation function considered Rectified Linear Unit (ReLU). After each convolutional layer, the Max Pooling operation is performed with a size of (2 × 2 × 2), which determines the highest value in segments of a feature map, and utilizes this information to generate a reduced (pooled) feature map. In order to compress the data between all convolution steps, Batch Normalization is carried out. As depicted in Fig. 3, the input [32, 32, 32, 1] undergoes compression within the encoder, resulting in a reduced size of [4, 4, 4, 64].

Subsequently, the data is flattened and substitutes the decoder with a trainable non-linear function, represented by the dense layers (located in the right section of Fig. 6). This function is responsible for transforming the encoded representation into scalar output values corresponding to the elasticity tensor. The model employs five dense layers with 32, 8, 8, 8, and 3 neurons in each respective layer each of them has the same activation function as the convolutional ones except the final dense layer does not have an activation function. The terminology utilized in the network for various operations, such as generating a convolution kernel (Conv3D) and downsampling data (MaxPool3D), follows the nomenclature introduced by PyTorch.

#### Neural network training

In the current work, the generated dataset is split into three sections of training, testing, and validation. We create 2572 random atomistic structures with a random percentage of vacancies for training. The test dataset is just employed to assess the network’s precision after training. Note that 10% of the training data is devoted to validating the network. There are 346835 parameters that can be trained in the neural network. During the training process, the network’s performance is checked on the validation dataset in order to prevent over- or under-fitting. Overall, the training is accomplished by minimization of loss or cost function. In this case, the mean squared error (MSE) loss function is applied. The MSE is a widely employed metric in multi-output regression tasks where the network predicts several different continuous values simultaneously. This evaluation metric operates by calculating the error for each output neuron, denoted as $\hat{Y}_{i,k}$, where $i$ indexes the data points, and $k$ indexes the output neurons (typically ranging from 1 to the total number of outputs, e.g., 1, 2, and 3 in our case). Specifically, for each output, the error is computed as the squared difference between the predicted value ($\hat{Y}_{i,k}$) and the actual value ($Y_{i,k}$). This squaring of errors serves a dual purpose: firstly, it places a greater penalty on larger errors, effectively emphasizing their significance; secondly, it ensures that negative and positive errors do not cancel each other out during the aggregation process. To assess the overall performance of our model, the sum of these squared errors across all output neurons is calculated for each data point, thereby yielding the total squared error for that particular data point across all outputs. Subsequently, we determine the average of these sums across all data points within our dataset. This computed average, depicted in Eq. (13), represents the final measure of the mean squared error across our entire dataset.

13 $$\begin{aligned} MSE = \frac{1}{n} \sum _{i=1}^{n} \sum _{k=1}^{s} (Y_{i,k} - \hat{Y}_{i,k})^2 \end{aligned}$$

here, $n$ is the number of data points, and the inner sum is over the $s$ output neurons

During the training phase of a neural network, Mean Squared Error (MSE) plays a crucial role as a loss function. The network’s primary objective is to adjust its weights and biases to minimize the MSE. This optimization is achieved through a process known as backpropagation. In backpropagation, the gradient of the MSE with respect to each weight and bias in the network is computed, allowing the network to understand how to adjust its parameters to reduce the error. In addition to measuring MSE during training, the network also evaluates MSE on a separate validation dataset. This step is critical for assessing the network’s generalization capabilities. By comparing the training and validation losses after each epoch, which is a complete pass through the entire training dataset, the network can evaluate its performance more holistically. This comparison serves as a vital metric for determining if the model is overfitting or underfitting. Overfitting occurs when the model performs well on the training data but poorly on unseen data, while underfitting is when the model performs poorly even on the training data. In our specific study, there are no significant indications of either overfitting or underfitting. This suggests that the model is appropriately capturing the underlying patterns in the data without being overly specific to the training dataset or too general and simplistic. Such a balance is essential for a model to perform well on both the training data and unseen data, indicating effective learning and generalization capabilities.

This particular loss function is usually coupled with an optimizer, whose primary role is to modify the model’s weights and biases according to the computed error. This operation is done using ADAM optimizer^57^ as a gradient descent algorithm with $1.0\times 10^{-4}$ learning rate. The arguments of $\beta _{1}$ = 0.9, $\beta _{2}$ = 0.999 and $\varepsilon$ = $10^{-7}$ are set as the other parameters of this optimizer. the training involves estimating the gradient using random samples in batches comprising 64 data, and it is repeated this process for 150 epochs.

### Supplementary Information

Supplementary Information.

## Data Availability

The data that support the findings of this study are available from the corresponding author upon reasonable request.

## Code availability

The code used for ML in this study is open-source and accessible in github.com/pytorch. The LAMMPS Molecular Dynamics Simulator is also open source and accessible at lammps.org. Other codes and scripts used in this work are available from the corresponding author upon reasonable request.
