# Supplementary material for: Rapid and accurate predictions of perfect and defective material properties in atomistic simulation using the power of 3D CNN-based trained artificial neural networks
Source: Sci Rep. 2024 Jan 2;14:36. doi: 10.1038/s41598-023-50893-9 (PMC10762098; doi:10.1038/s41598-023-50893-9)
Supplement: Supplementary file 1 — Supplementary Information. [file 41598_2023_50893_MOESM1_ESM.pdf]

# Supplementary Information

# Rapid and Accurate Predictions of Perfect and Defective Material Properties in Atomistic Simulation Using the Power of 3D CNN-Based Trained Artificial Neural Networks

**Iman Peivaste[1,2], Saba Ramezani[1], Ghasem Alahyarizadeh[1,*], Reza Ghaderi[3], Ahmed Makradi[2], and Salim Belouettar[2]**

[1]Faculty of Engineering, Shahid Beheshti University, Tehran, Iran
[2]Luxembourg Institute of Science and Technology, 5, avenue des Hauts-Fourneaux, L-4362 Esch-sur-Alzette, Luxembourg
[3]Department of Electrical Engineering, Shahid Beheshti University, Tehran, Iran
[*]Corresponding Author:g_alahyarizadeh@sbu.ac.ir

## Supplementary Methods

### Introduction

The methodologies for calculating mechanical properties from elastic constants are presented in this section, as derived from reference[1]. Interested readers are encouraged to consult this study for more in-depth information. We have tabulated the calculated properties obtained from both Molecular Dynamics (MD) and Machine Learning (ML) methods, utilizing trained Artificial Neural Networks (tANNs), in tables 1 to 4. These tables provide a comprehensive overview of the properties we have computed and are presented for reference in this section.

### Elastic and compliance constants

In accordance with Hook's Law, equation 1 in its tensorial form can describe the connection between stress and strain in the context of small displacements, commonly referred to as Voigt notation. In this notation, $C_{ij}$ represents the elastic constant, and both stress and strain are each represented as six-component tensors.

$$\sigma_i = \sum_{1}^{6} C_{ij}\varepsilon_{ij} \tag{1}$$

In certain elasticity studies, it is advantageous to define strains in terms of stresses. Consequently, we derive equation 2, in which $S_{ij}$ represents the elastic compliance constants.

$$\varepsilon_i = \sum_{1}^{6} S_{ij}\sigma_j \tag{2}$$

By employing the principles of continuous elastic theory, equations 1 and 2 can be adapted for triclinic crystals, resulting in 16 independent constants. However, in the case of cubic crystals, the equations are simplified due to geometric symmetry, and each tensor can be expressed using only three independent constants. Generally, these reduced equations are written as follows:

$$C = \begin{bmatrix} C_{11} & C_{12} & 0 & 0 \\ C_{21} & C_{22} & 0 & 0 \\ 0 & 0 & 0 & 0 \\ 0 & 0 & 0 & C_{44} \end{bmatrix} \tag{3}$$

$$S = \begin{bmatrix} S_{11} & S_{12} & 0 & 0 \\ S_{21} & S_{22} & 0 & 0 \\ 0 & 0 & 0 & 0 \\ 0 & 0 & 0 & S_{44} \end{bmatrix} \tag{4}$$

Equations 5-7 reveal the relationships between elastic and compliance constants:

$$S_{11} = \frac{C_{11} + C_{12}}{(C_{11} - C_{12})(C_{11} + 2C_{12})} \tag{5}$$

$$S_{12} = \frac{-C_{12}}{(C_{11} - C_{12})(C_{11} + 2C_{12})} \tag{6}$$

$$S_{44} = \frac{1}{C_{44}} \tag{7}$$

## Mechanical properties

The mechanical properties of cubic systems, including the bulk modulus, shear modulus, and young's modulus, can be calculated using the Voigt-Reuss-Hill scheme. This scheme employs the following equations 8-12, where H, V, and R represent the Hill, Voigt, and Reuss approximations, respectively.

$$B_H = B_V = B_R = \frac{C_{11} + 2C_{12}}{3} \tag{8}$$

$$G_H = \frac{G_V + G_R}{2} \tag{9}$$

$$G_V = \frac{C_{11} - C_{12} + 3C_{44}}{5} \tag{10}$$

$$G_R = \frac{5(C_{11} - C_{12})C_{44}}{4C_{44} + 3(C_{11} - C_{12})} \tag{11}$$

$$E_H = \frac{9B_H G_H}{3B_H + G_H} \tag{12}$$

(For simplicity, hereafter, variables without subscripts represent Hill approximation.)

## Poisson's and Pugh ratios

Poisson's and Pugh's ratios are two mechanical parameters of the material that are determined based on the values of B and G, as expressed in equations (13) and (14). These parameters serve as indicators of the material's ductility.

$$v = \frac{3B - 2G}{2(3B + G)} \tag{13}$$

$$Pugh\ ratio = \frac{B}{G} \tag{14}$$

Poisson's ratio is introduced as a metric to evaluate the shear resistance of crystals, where a higher value signifies improved material plasticity. Notably, a threshold value of 0.26 serves as a critical point, designating materials with a Poisson's ratio exceeding this threshold as ductile and less brittle in nature. Furthermore, Poisson's ratio offers valuable insights into the interatomic forces acting within materials. When Poisson's ratio falls within the range of 0.25 to 0.5, it signifies that the interatomic forces within the solid are predominantly central forces. However, for values outside this range, interatomic forces are categorized as non-central forces.

Pugh's ratio serves as a quantitative indicator of a material's brittleness or ductility. The pivotal threshold for this parameter is typically set at 1.75, with materials possessing a Pugh's ratio exceeding this value classified as ductile, while those falling below it are deemed brittle.

## Cauchy pressure

In accordance with equation (15), the Cauchy Pressure stands as an additional parameter that offers insights into the brittleness or ductility characteristics of the material.

$$C" = C_{12} - C_{44} \tag{15}$$

A positive Cauchy pressure reading signifies that the material exhibits ductile behavior, displaying characteristics associated with metallic properties and primarily metallic atom-to-atom bonding.

## Longitudinal Modulus

The longitudinal elastic modulus, also referred to as the pressure wave (P wave) modulus, assesses the response of an isotropic elastic material to linear stress conditions under uniaxial strain. Equation (16) outlines this modulus.

$$M = \frac{3B+4G}{3} \quad (16)$$

## Hardness

Hardness serves as a crucial material parameter, particularly in the evaluation of wear properties within material structures. In microscopic assessments, various methods are employed to calculate hardness, one of which is known as Vickers hardness. There are two models for determining Vickers hardness, established by Tian ($H_{V1}$) and Chen ($H_{V2}$). These models are defined based on bulk modulus and shear modulus, as outlined in equations (17-19).

$$k = \frac{G}{B} \quad (17)$$

$$H_{V1} = 0.92k^{1.137}G^{0.708} \quad (18)$$

$$H_{V2} = 2\left(k^2G\right)^{0.585} - 3 \quad (19)$$

An alternative relationship for calculating microhardness ($H_m$) is established using Young's modulus and Poisson"s ratio, as defined in equation (20).

$$H_m = \frac{(1-2\nu)E}{6(1+\nu)} \quad (20)$$

## Elastic anisotropy

In reality, the elastic behavior of crystals is often not isotropic, necessitating a meaningful index to quantify the extent of anisotropy. The term used for this specific purpose is "elastic anisotropy," which characterizes the nature of interatomic bonds in diverse crystallographic directions. This parameter holds significant importance, particularly in its association with the likelihood of micro-crack formation within solid materials. Consequently, assessing the elastic anisotropy of a crystal under varying conditions becomes crucial, as it can provide valuable insights for enhancing its mechanical durability.

In our study, we have employed a diverse set of methods to ascertain and assess the elastic anisotropy. One such method is the Zerner anisotropy, which quantifies the level of anisotropy by considering atomic bonds across distinct crystal planes. For BCC crystals, this anisotropy measure is computed using the following equation.

$$A_1 = A_2 = A_3 = \frac{2C_{44}}{C_{11}-C_{12}} \quad (21)$$

In the case of a completely elastic material, the values of $A_1$, $A_2$, and $A_3$ are all equal and set to one. Any value exceeding or falling below one indicates the degree of anisotropy in the respective crystal plane.

a) Compressibility and Shear anisotropy

The compressibility factor (A_B) and shear anisotropy factor (A_G) for a crystal, which are denoted as the Chung-Buessem empirical anisotropy index, can be determined using equations (22) and (23), respectively:

$$A_B = \frac{B_V - B_R}{B_V + B_R} \quad (22)$$

$$A_G = \frac{G_V - G_R}{G_V + G_R} \quad (23)$$

Where *V* and *R* subscripts represented the Voight and Reuss approximation.

b) Universal anisotropy

Another index used to quantify the anisotropy of materials is the Universal anisotropy ($A^U$) which is defined in equation (24) based on the bulk and shear moduli of the materials:

$$A^U = \frac{B_V}{B_R} + 5\frac{G_V}{G_R} - 6 \tag{24}$$

c) Absolute anisotropy

The preceding indices presented for calculating materials' anisotropy are relative measures intended to assess the crystal's anisotropy. This means that a material with $A^U = 3$ does not necessarily possess an anisotropy level twice that of a material with $A^U = 1.5$. To address this limitation, Kube introduced an absolute anisotropy index, which quantifies anisotropy by computing the log-Euclidean distance between $C_V$ and $C_R$, representing the stiffness tensors obtained from Voigt's and Reuss's approximations, as expressed in Equation (25):

$$A^L = \sqrt{\left[\ln\left(\frac{B_V}{B_R}\right)\right]^2 + 5\left[\ln\left(\frac{G_V}{G_R}\right)\right]^2} \tag{25}$$

For isotropic materials, the values of $A_B$, $A_G$, $A^U$ and $A^L$ should be null. Any deviation from zero indicates the degree of anisotropy present in the material. Notably, in the case of cubic crystals where $B_V$ is equal to $B_R$, $A_B$ is indeed equal to zero. Furthermore, $A_G$, $A^U$, and $A^L$ in cubic crystals are solely dependent on the shear modulus.

## Mechanical properties on crystallography planes

Materials with a crystalline structure consist of multiple atomic planes that exhibit periodic alignment. To gain a more comprehensive understanding of the elastic anisotropy in metals, it is essential to predict the directional dependence of moduli. This approach enables us to assess the crystal's response within each specific plane, thereby enhancing our insight into its anisotropic behavior.

In the context of a cubic crystal, there exist three principal directions: <100>, <110>, and <111>. The moduli associated with each of these directions can be estimated using the elastic or compliance constants. However, for a Body-Centered Cubic (BCC) crystal, it is important to note that the bulk modulus does not exhibit any dependency on the crystal's directional orientation.

The shear moduli along the principal directions <100>, <110>, and <111> are derived by Equations (26-28). These equations rely on the calculated elastic constants, with $G_{100}$ representing the shear modulus in the <100> direction on {010} plane, $G_{110}$ which is normally known as the shear constant represents the shear modulus along the <110> direction on {110} plane and $G_{111}$ indicates to the shear modulus in <111> direction along the {011}, {112} or {123} planes:

$$G_{100} = C_{44} \tag{26}$$

$$G_{110} = \frac{C_{11} - C_{12}}{2} \tag{27}$$

$$G_{111} = \frac{3(C_{11} - C_{12})C_{44}}{C_{11} - C_{12} + 4C_{44}} \tag{28}$$

The resistance of a crystal to compressive or tensile stresses applied in a particular plane is characterized by the directional Young's modulus. In the case of a Body-Centered Cubic (BCC) crystal, the Young's modulus for the three low-index planes {100}, {110}, and {111}, along the normal directions <100>, <110>, and <111>, is represented by $E_{100}$, $E_{110}$, and $E_{111}$, respectively. These modulus values are determined in terms of elastic compliance constants and are defined in equations (29-31):

$$E_{100} = \frac{1}{S_{11}} \tag{29}$$

$$E_{110} = \frac{4}{2S_{11} + 2S_{12} + S_{44}} \tag{30}$$

$$E_{111} = \frac{3}{S_{11} + 2S_{12} + S_{44}} \tag{31}$$

The anisotropy of Poisson’s ratio in arbitrary directions along three low-index planes of {100}, {110}, and {111} are named $\nu_{100}$, $\nu_{110}$, and $\nu_{111}$, respectively. These anisotropic Poisson's ratios for cubic crystals are defined by equations (32-34):

$$\nu_{100} = -\frac{S_{12}}{S_{11}} \tag{32}$$

$$\nu_{110} = -\frac{2S_{11} + 6S_{12} - 2S_{44}}{4S_{11} + 4S_{12} + 2S_{44}} \tag{33}$$

$$\nu_{111} = -\frac{2S_{11} + 4S_{12} - S_{44}}{2S_{11} + 4S_{12} + 2S_{44}} \tag{34}$$

## Supplementary tables

| | | Elastic Constants | | | Bulk Modulus | Shear Modulus | | | Young Modulus | Longitudial Modulus |
|---|---|---|---|---|---|---|---|---|---|---|
| Percentage | Method | C11 (GPa) | C12 (GPa) | C44 (GPa) | B (GPa) | Gv (GPa) | Gr (GPa) | Gh (GPa) | E (GPa) | B+(4/3)E (GPa) |
| 0 | MD | 224.03 | 137.610 | 119.964 | 166.42 | 89.26 | 70.14 | 79.70 | 206.19 | 272.69 |
| | tANNs | 223.41 | 137.340 | 119.560 | 166.03 | 88.95 | 69.87 | 79.41 | 205.47 | 271.91 |
| 1 | MD | 217.12 | 133.224 | 114.633 | 161.19 | 85.56 | 67.71 | 76.63 | 198.45 | 263.37 |
| | tANNs | 216.73 | 132.950 | 114.400 | 160.88 | 85.40 | 67.60 | 76.50 | 198.09 | 262.87 |
| 2 | MD | 210.29 | 128.746 | 109.399 | 155.93 | 81.95 | 65.38 | 73.67 | 190.93 | 254.15 |
| | tANNs | 209.99 | 128.430 | 109.110 | 155.62 | 81.78 | 65.33 | 73.55 | 190.62 | 253.69 |
| 3 | MD | 203.58 | 124.380 | 104.287 | 150.78 | 78.41 | 63.07 | 70.74 | 183.53 | 245.10 |
| | tANNs | 203.22 | 123.760 | 104.030 | 150.25 | 78.31 | 63.15 | 70.73 | 183.41 | 244.55 |
| 4 | MD | 196.97 | 119.744 | 99.180 | 145.49 | 74.95 | 60.95 | 67.95 | 176.39 | 236.09 |
| | tANNs | 197.4 | 119.380 | 98.860 | 145.39 | 74.92 | 61.26 | 68.09 | 176.69 | 236.18 |
| 5 | MD | 189.96 | 115.467 | 94.086 | 140.30 | 71.35 | 58.43 | 64.89 | 168.67 | 226.82 |
| | tANNs | 189.65 | 115.170 | 93.940 | 140.00 | 71.26 | 58.38 | 64.82 | 168.46 | 226.43 |
| 6 | MD | 183.40 | 110.997 | 89.293 | 135.13 | 68.06 | 56.28 | 62.17 | 161.71 | 218.02 |
| | tANNs | 183.12 | 110.780 | 89.068 | 134.89 | 67.91 | 56.19 | 62.05 | 161.41 | 217.63 |
| 7 | MD | 176.22 | 106.218 | 84.637 | 129.55 | 64.78 | 54.01 | 59.39 | 154.56 | 208.75 |
| | tANNs | 175.91 | 106.040 | 84.386 | 129.33 | 64.61 | 53.88 | 59.24 | 154.18 | 208.32 |
| 8 | MD | 169.08 | 101.986 | 80.262 | 124.35 | 61.58 | 51.55 | 56.56 | 147.35 | 199.77 |
| | tANNs | 168.85 | 101.910 | 80.021 | 124.22 | 61.40 | 51.42 | 56.41 | 146.98 | 199.43 |
| 9 | MD | 160.97 | 95.916 | 74.625 | 117.60 | 57.79 | 49.17 | 53.48 | 139.32 | 188.90 |
| | tANNs | 160.71 | 95.756 | 74.137 | 117.41 | 57.47 | 49.00 | 53.23 | 138.74 | 188.39 |
| 10 | MD | 154.34 | 91.972 | 70.409 | 112.76 | 54.72 | 46.84 | 50.78 | 132.46 | 180.47 |
| | tANNs | 154.08 | 91.812 | 70.187 | 112.56 | 54.56 | 46.73 | 50.65 | 132.13 | 180.10 |

**Table 1.** the calculated properties obtained from both MD and ML methods including Elastic constants and moduli

| | | Poisson Ratio | | Cauchy Press. | Hardness | | | Shear Constant | Machinability Index |
|---|---|---|---|---|---|---|---|---|---|
| Percentage | Method | $\nu$ | Pugh Ratio | C" (GPa) | H(v1) | H(v2) | H(m) | C' (GPa) | B/C44 |
| 0 | MD | 0.2935 | 2.088 | 17.65 | 8.84 | 7.95 | 10.97 | 43.21 | 1.39 |
| | tANNs | 0.2937 | 2.091 | 17.78 | 8.80 | 7.91 | 10.92 | 43.04 | 1.39 |
| 1 | MD | 0.2948 | 2.103 | 18.59 | 8.53 | 7.61 | 10.48 | 41.95 | 1.41 |
| | tANNs | 0.2948 | 2.103 | 18.55 | 8.52 | 7.60 | 10.47 | 41.89 | 1.41 |
| 2 | MD | 0.2959 | 2.117 | 19.35 | 8.23 | 7.29 | 10.02 | 40.78 | 1.43 |
| | tANNs | 0.2958 | 2.116 | 19.32 | 8.23 | 7.28 | 10.01 | 40.78 | 1.43 |
| 3 | MD | 0.2971 | 2.131 | 20.09 | 7.94 | 6.97 | 9.57 | 39.60 | 1.45 |
| | tANNs | 0.2965 | 2.124 | 19.73 | 7.97 | 7.01 | 9.59 | 39.73 | 1.44 |
| 4 | MD | 0.2979 | 2.141 | 20.56 | 7.67 | 6.68 | 9.15 | 38.62 | 1.47 |
| | tANNs | 0.2974 | 2.135 | 20.52 | 7.71 | 6.73 | 9.19 | 39.01 | 1.47 |
| 5 | MD | 0.2996 | 2.162 | 21.38 | 7.35 | 6.32 | 8.67 | 37.25 | 1.49 |
| | tANNs | 0.2994 | 2.160 | 21.23 | 7.35 | 6.32 | 8.67 | 37.24 | 1.49 |
| 6 | MD | 0.3006 | 2.174 | 21.70 | 7.08 | 6.03 | 8.27 | 36.20 | 1.51 |
| | tANNs | 0.3006 | 2.174 | 21.71 | 7.07 | 6.02 | 8.25 | 36.17 | 1.51 |
| 7 | MD | 0.3012 | 2.181 | 21.58 | 6.83 | 5.76 | 7.87 | 35.00 | 1.53 |
| | tANNs | 0.3013 | 2.183 | 21.65 | 6.81 | 5.74 | 7.85 | 34.94 | 1.53 |
| 8 | MD | 0.3025 | 2.198 | 21.72 | 6.54 | 5.43 | 7.45 | 33.55 | 1.55 |
| | tANNs | 0.3028 | 2.202 | 21.89 | 6.51 | 5.40 | 7.42 | 33.47 | 1.55 |
| 9 | MD | 0.3026 | 2.199 | 21.29 | 6.28 | 5.16 | 7.04 | 32.53 | 1.58 |
| | tANNs | 0.3031 | 2.205 | 21.62 | 6.24 | 5.11 | 6.99 | 32.48 | 1.58 |
| 10 | MD | 0.3042 | 2.220 | 21.56 | 5.99 | 4.82 | 6.62 | 31.18 | 1.60 |
| | tANNs | 0.3043 | 2.222 | 21.62 | 5.97 | 4.80 | 6.60 | 31.13 | 1.60 |

**Table 2.** the calculated properties obtained from both MD and ML methods including mechanical behaviours

| | | Compliance Elastic Constants | | | Mechanical Properties | | | | | |
|---|---|---|---|---|---|---|---|---|---|---|
| Percentage | Method | S11 (1/GPa) | S12 (1/GPa) | S44 (1/GPa) | G (100) (GPa) | G(110) (GPa) | G(111) (GPa) | E(100) (GPa) | E(110) (GPa) | E(111) (GPa) |
| 0 | MD | 0.00838 | -0.0031 | 0.0083 | 119.96 | 43.21 | 54.93 | 119.31 | 213.67 | 290.17 |
| | tANNs | 0.00841 | -0.00320 | 0.00836 | 119.56 | 43.04 | 54.71 | 118.84 | 212.92 | 289.25 |
| 1 | MD | 0.00863 | -0.0032 | 0.00872 | 114.63 | 41.95 | 53.19 | 115.80 | 205.90 | 278.00 |
| | tANNs | 0.00864 | -0.00328 | 0.00874 | 114.40 | 41.89 | 53.11 | 115.63 | 205.54 | 277.44 |
| 2 | MD | 0.00888 | -0.0033 | 0.0091 | 109.40 | 40.78 | 51.56 | 112.52 | 198.36 | 265.99 |
| | tANNs | 0.00888 | -0.00337 | 0.00916 | 109.11 | 40.78 | 51.54 | 112.51 | 198.07 | 265.32 |
| 3 | MD | 0.00915 | -0.0034 | 0.0095 | 104.29 | 39.60 | 49.92 | 109.24 | 190.89 | 254.24 |
| | tANNs | 0.00912 | -0.00345 | 0.00961 | 104.03 | 39.73 | 50.04 | 109.54 | 190.83 | 253.57 |
| 4 | MD | 0.00939 | -0.0035 | 0.0100 | 99.18 | 38.62 | 48.48 | 106.43 | 183.74 | 242.45 |
| | tANNs | 0.00930 | -0.00350 | 0.01011 | 98.86 | 39.01 | 48.87 | 107.42 | 184.19 | 241.78 |
| 5 | MD | 0.00974 | -0.0036 | 0.0106 | 94.09 | 37.25 | 46.64 | 102.67 | 175.86 | 230.69 |
| | tANNs | 0.00974 | -0.00368 | 0.01064 | 93.94 | 37.24 | 46.62 | 102.62 | 175.66 | 230.31 |
| 6 | MD | 0.01002 | -0.0037 | 0.0111 | 89.29 | 36.20 | 45.15 | 99.71 | 168.81 | 219.53 |
| | tANNs | 0.01003 | -0.00378 | 0.01122 | 89.07 | 36.17 | 45.10 | 99.61 | 168.51 | 219.00 |
| 7 | MD | 0.01038 | -0.0039 | 0.0118 | 84.64 | 35.00 | 43.51 | 96.33 | 161.49 | 208.51 |
| | tANNs | 0.01040 | -0.00391 | 0.01185 | 84.39 | 34.94 | 43.42 | 96.15 | 161.11 | 207.93 |
| 8 | MD | 0.01082 | -0.0040 | 0.0124 | 80.26 | 33.55 | 41.62 | 92.34 | 154.03 | 198.15 |
| | tANNs | 0.01085 | -0.00408 | 0.01249 | 80.02 | 33.47 | 41.52 | 92.14 | 153.65 | 197.63 |
| 9 | MD | 0.01119 | -0.0041 | 0.0134 | 74.63 | 32.53 | 40.06 | 89.34 | 145.84 | 184.79 |
| | tANNs | 0.01121 | -0.00418 | 0.01348 | 74.14 | 32.48 | 39.96 | 89.21 | 145.26 | 183.74 |
| 10 | MD | 0.01167 | -0.0043 | 0.0142 | 70.409 | 31.18 | 38.29 | 85.66 | 138.73 | 174.83 |
| | tANNs | 0.01169 | -0.00436 | 0.01424 | 70.18 | 31.13 | 38.22 | 85.51 | 138.39 | 174.32 |

**Table 3.** the calculated properties obtained from both MD and ML methods including mechanical properties on crystallography planes

| | | Zener anisotropy factor | Shear anisotropy | Universal anisotropy | Absolute anisotropy |
|---|---|---|---|---|---|
| Percentage | Method | A(z) | A(G) | A(u) | A(L) |
| 0 | MD | 2.7761 | 0.1199 | 1.3636 | 0.5392 |
| | tANNs | 2.7782 | 0.1201 | 1.3657 | 0.5400 |
| 1 | MD | 2.7326 | 0.1164 | 1.3183 | 0.5232 |
| | tANNs | 2.7309 | 0.11633 | 1.3165 | 0.5226 |
| 2 | MD | 2.6828 | 0.1124 | 1.2667 | 0.504 |
| | tANNs | 2.6755 | 0.1118 | 1.2591 | 0.5022 |
| 3 | MD | 2.6334 | 0.1084 | 1.2158 | 0.4867 |
| | tANNs | 2.6184 | 0.1071 | 1.2004 | 0.4811 |
| 4 | MD | 2.5683 | 0.1030 | 1.1492 | 0.4626 |
| | tANNs | 2.5342 | 0.10028 | 1.1145 | 0.4499 |
| 5 | MD | 2.5257 | 0.0995 | 1.1060 | 0.4468 |
| | tANNs | 2.5225 | 0.0993 | 1.1027 | 0.4456 |
| 6 | MD | 2.4663 | 0.0947 | 1.0462 | 0.4248 |
| | tANNs | 2.4624 | 0.0943 | 1.0422 | 0.4233 |
| 7 | MD | 2.4179 | 0.0907 | 0.9978 | 0.406 |
| | tANNs | 2.4155 | 0.0905 | 0.9954 | 0.4059 |
| 8 | MD | 2.3923 | 0.0886 | 0.972 | 0.3973 |
| | tANNs | 2.3908 | 0.0884 | 0.9709 | 0.3968 |
| 9 | MD | 2.2942 | 0.0805 | 0.8761 | 0.3610 |
| | tANNs | 2.2827 | 0.0796 | 0.8649 | 0.3567 |
| 10 | MD | 2.2576 | 0.0775 | 0.8407 | 0.3475 |
| | tANNs | 2.2543 | 0.0772 | 0.8375 | 0.3462 |

**Table 4.** the calculated properties obtained from both MD and ML methods including anisotropy factors

## References

1. Alivaliollahi, A., Alahyarizadeh, G. & Minuchehr, A. Effect of temperature, pressure, crystal defect types, and densities on the mechanical behavior of tungsten under tensile deformation: A molecular dynamics simulation study. *Nucl. Mater. Energy* 101555 (2023).
